# Medical Photography Usage Amongst Doctors at a Portuguese Hospital

**DOI:** 10.3390/ijerph19127304

**Published:** 2022-06-14

**Authors:** Mariana Cura, Hélio Alves, José Paulo Andrade

**Affiliations:** 1 Faculty of Medicine, University of Porto, 4200-319 Porto, Portugal; up201605072@med.up.pt; 2 Unit of Anatomy, Department of Biomedicine, Faculty of Medicine, University of Porto, Alameda Professor Hernâni Monteiro, 4200-319 Porto, Portugal; helioalves@med.up.pt; 3 CINTESIS@RISE, Faculty of Medicine, University of Porto, 4200-450 Porto, Portugal

**Keywords:** photography, clinical photography, medical technologies, questionnaire

## Abstract

Technological advancements in smartphones have made it possible to create high-quality medical photographs, with the potential to revolutionise patient care. To ensure the security of the patient’s data, it is important that medical professionals receive informed consent from the patient, that physical conditions are met to take a photograph, and that these medical images are stored correctly. This study aimed to determine if medical professionals of an academic hospital make use of medical photography, and how the content is obtained, stored, transferred, and used. **Methods:** A 30-question questionnaire was distributed across 29 medical departments at Centro Hospitalar Universitário de São João (CHUSJ), a tertiary referral and teaching hospital in Porto, Portugal, with approximately 900 medical professionals. Quantitative statistical methods were used to analyse questionnaire responses. **Results:** There were a total of 257 respondents. Of these, 93% used medical photography, 70% used it to document a patient’s clinical progress, 70% to ask for a second opinion, 56% for education, 65% for research and publication, and 68% to present at medical conferences. Medical photography was used by 33% weekly and 36% monthly, with 71% of respondents always asking for the patients’ consent before taking a photograph. Doctors aged 20–40 years used photography more often than doctors over 40 years of age to document the clinical progress of the patients (77% and 52%, respectively, *p* = 0.01) and to ask for a second opinion (78% and 52%, respectively, *p* < 0.001). **Conclusions:** Our study shows that medical photography is a common practice amongst medical doctors. However, appropriate measures need to be created to obtain patients’ consent, store images, and sure the security of patients’ information.

## 1. Background

Before the invention of the modern photograph, medical professionals could only rely on drawings and descriptions written in books to learn the physical presentations of syndromes and diseases [1]. They also needed to rely only on their memory and written accounts to recall their patients’ presentations. This all changed with the arrival of photography, and now photographs are essential to illustrate clinical findings, operative steps, or surgical results. These images are also important for medical records, medical education, and to seek advice from colleagues. It is important to note that lectures, scientific communications, posters, thesis, and dissertations also require photographs, in order to substantiate their research value [2].

More recently, high-speed data connections and high-quality cameras in smartphones have made it possible not only to create high-quality images in seconds, without the extensive training historically required to operate a high-resolution digital camera [3], but also to allow fast collaboration between clinicians by sharing medical photographs of patients [4].

These technological advancements have unleashed medical professionals’ ability to document a patient’s clinical progression, including details for wound documentation, for example, to avoid repeated medical examinations, and seeing wound progression over time [5,6]. In medical fields that rely heavily on visual physical examinations, such as plastic surgery [7], maxillo-facial surgery [8], and orthopaedic surgery [9], medical photography can also significantly help patient care [10,11]. Among dermatologists that frequently inspect suspicious lesions, one study found that 89% of the respondents agree that medical photography facilitates patient care [12]. Another study conducted amongst dermatologists in the USA showed that these medical professionals commonly use medical photography to document biopsy sites; to monitor disease progression; for education, research, and publication; and to ask for a second opinion [13]. It is important to note that these practices can affect patients, with a different study in the USA on medical photography revealing that patients are concerned about data confidentiality (65.5%), protection of the photographs (56.8%), automatic upload to a cloud server (33.8%), and professionalism (31.5%). Around 20% had no concerns regarding the usage of a smartphone camera for medical photography [14].

For the purposes of this study, we defined medical photography as the documentation of a clinical appearance of a patient in any context, which includes, but is not limited to, clinical appointments, hospitalisation, surgical procedures, pathology, and legal responsibilities. Before taking photographs, the medical professional needs to ensure that various conditions are met. The first is the consent of the patient, the second is the ideal physical conditions to take the photograph, the third is the storage location of the photographs, and the fourth is the security of access to the images obtained [15].

Despite the widespread use of medical photography, it is not yet known how physicians of different specialties in a teaching hospital use images in their clinical practice, how they deal with the issue of patient consent, and how they tackle problems related to privacy, security, and image storage. This is particularly the case given current general and health-specific privacy laws; these include the General Data Protection Regulation (GDPR) of the European Union (EU), which is applicable in all member states of the European Union as of the 25th of May 2018 [16].

Existing papers generally consider physicians of one specialty [6,7,10,11,13,17,18], and studies undertaken at a national level represent a relatively low number of respondents considering the potential total number [6,7,10,11,13]. There are also no studies in this area performed in the EU since the implementation of the GDPR.

Therefore, the main objective of the present work was to determine the prevalence of the use of medical photography among the medical doctors of a tertiary teaching hospital in Portugal. In addition, we hoped to gain some information about how medical doctors (specialists and residents) of several specialties use medical images and how the content is obtained, stored, and transferred.

## 2. Methods

A 30-question questionnaire was devised based on the precedent set by a previous study [13], Institute of Medical Illustrators (IMI) guidelines [19], and the GDPR [16]. It was divided into five sections: sociodemographic characteristics (questions (Q) 1–3); medical photography usage and frequency (Q 4–6, 22–26); technical aspects (Q 7–9, 15–17); consent (Q 10–14); and security, storage, and transfer (Q 18–21, 27–30). Data collected in some questions are not discussed in this paper, as they are not within the objectives of the present study (i.e., they mainly relate to technical aspects). The questionnaire was distributed across 29 medical departments of the Centro Hospitalar Universitário de São João (CHUSJ), a tertiary referral teaching hospital in Porto, Portugal, with approximately 900 medical professionals. The study was analysed, reviewed, and approved by the Ethics Committee of the CHUSJ and the Faculty of Medicine of the University of Porto. The questionnaire was written in Portuguese and distributed from March 2021 to November 2021 through an email sent by the medical department director with a link to an online questionnaire, or by paper during a department meeting. An English translated copy can be found in Supplementary Material S1. During data collection, all answers were anonymised.

Descriptive statistics of the most relevant questions for the aims of this paper are presented as absolute counts and proportions. Medical specialties were grouped into three types—medical, medical–surgical, and surgical—based on their professional activity and to easily compare and interpret data depending on their fields (see Supplementary Material S2 for the discrimination of the categories). The practitioner levels included residents (medical in training) and specialists (all levels of specialist practitioners). Since several questions allowed the participants to select more than one answer, each of those possible answers was treated as a dichotomous variable (yes/no). In questions that had several answer options and only allowed the selection of one of those, re-categorisation of the answers was *a priori* decided for the purposes of hypothesis testing, to avoid excessive dispersion and under-representativeness of each category (for example, Q 5 regarding the frequency of photography use in clinical practice allowed 6 possible answers—several times a day, daily, weekly, monthly, annually, never—that were to be dichotomously categorised as “weekly or more frequently” and “monthly or less frequently” for analysis purposes; similar planned decisions were made regarding Q 8 and Q 13).

Key questions regarding the reason for the use of medical photography (Q 4), the frequency of its use (Q 5), the nature of the device on which they were taken (Q 8), and whether written or oral consent was obtained (Q 11 and Q 12), where that consent was stored (Q 13), and if a new consent was sought when re-using the photograph for other intentions (Q 27 and Q 28), were analysed according to the sociodemographic variables to explore possible differences between groups. The chi-square test (or, when appropriate, Fisher’s exact test) was applied, and the significance level was set at α = 0.01.

The online data collection was performed using Typeform (online survey platform, TYPEFORM SL, Barcelona, Spain), the data was stored using an encrypted, data-loss preventive file system, and data management and analysis were undertaken using Microsoft Excel (Office 365, year 2022, version 2202. Microsoft Corporation, Redmond, WA, USA) and Statistical Package for the Social Sciences (SPSS) (IBM Corp. Released 2019. IBM SPSS Statistics for Windows, Version 26.0. Armonk, NY, USA: IBM Corp.).

## 3. Results

A total of 257 respondents to the questionnaire was registered, corresponding to approximately 29% of the 900 doctors working at CHUSJ.

The sociodemographic characteristics of the study sample are presented in Table 1. Slightly more than half of the participants were female, specialists, and working in a medical specialty. The majority of the sample (70%) was 40 or less years old.

A relatively small proportion (7%) of the doctors reported never photographing their patients in their daily practice (Q 4.1), which was more common among older doctors (15% in those above 40 years old and 4% with ages between 20 and 40, *p* = 0.003) (Table 2).

Considering the reasons to use photography (Q 4), the most common were to ask for a second opinion and to document the patient’s clinical progress (70%), to present at medical conferences (68%), for research and publication (65%), or educational purposes (56%). In comparison, legal purposes (9%), registering biopsy sites (7%), and pathology reasons (4%) were the least frequent reasons.

These reasons invoked to take photographs of patients were not uniform across groups (Table 2): younger doctors (77% vs. 52%, *p* < 0.001), and those in surgical and medical-surgical specialties (80% and 81% vs. 62%), seemed to be more disposed to using photography to document patients’ clinical progress, and younger doctors were also significantly more prone to use it to ask for second opinions (78% vs. 52%, *p* < 0.001) or for presentations at medical conferences (73% vs. 53%, *p* = 0.002).

When respondents were asked about the frequency with which they used medical photography, 33% said they used it on a weekly basis and 36% used it monthly. After associating all the frequencies, we found that 45% of the participants used it on a weekly or more frequent basis, and 55% used it monthly or less often (Q 5), with a significantly higher proportion of doctors of surgical and medical–surgical specialties (57% and 62%) using it weekly or more frequently than their counterparts in medical specialties (33%, *p* < 0.001).

The doctor’s personal device (Q 8), as opposed to an institutional device (8%) or a personal device only used for professional purposes (8%), was used by the vast majority (84%) of the participants, and that habit was significantly more prevalent in residents (92% vs. 78%, *p* = 0.003) and younger clinicians (89% vs. 71%, *p* = 0.001). Around 93% reported using a phone camera as the means to capture the photographs (Q 7).

Most respondents who take medical photographs stored them (Q 18) in the same device with which the photograph was taken (58%), followed by 38% in a personal device, 11% in a hospital device, 11% in a personal cloud, 10% in a personal hard drive, 9% in a personal device only for professional purposes, 9% in a patients’ digital file, 6% in a personal hard drive only for professional purposes, 6% in a hospital server, 4% in a hospital hard drive, and 3% in a personal cloud only for professional purposes.

When using a medical photograph to ask a colleague for a second opinion (Q 22), methods of communication varied amongst the respondents: WhatsApp (56%), personal email (34%), and institutional email (22%) were reported as the most used means, whereas showing the photograph in person (7%), and using Facebook Messenger (5%) or Short Message Service (SMS) (4%), were rarely reported.

Nearly 79% of all the respondents had received photographs when asked for a second opinion (Q 23), and similar patterns of means of communication were used: WhatsApp (66%), personal email (31%), institutional email (15%), being shown the photograph in person (7%), Facebook Messenger (10%), and SMS (7%).

Regarding informed consent, most doctors (71%) always asked for patients’ consent before taking the photograph (Q 10), but some rarely (6%) or never (4%) asked for it. However, 20% of the participants said they do not document the obtained oral or written consent (Q 13), and this practice was significantly more frequent in surgical and medical–surgical specialties (38% and 29%) than in medical specialties (12%, *p* = 0.002).

Table 3 describes the type of consent—oral (Q 11) or written (Q 12)—gained from the patients by the participants who used medical photography for specific reasons (as indicated in Q 4; for example, among those who, in Q 4, stated they usually took photographs for educational purposes, 50% reported they obtained verbal consent for that purpose in Q 11, 6% reported they obtained written consent for the same purpose in Q 12, 17% obtained both forms of consent, and 27% did not select any form of consent in Q 11 or Q 12).

It is clear that doctors who use photography for research and publication or to present in medical conferences resort to more formal types of consent, using written or oral and written consent much more frequently than when the photograph is taken for strictly clinical reasons (documenting clinical progress or asking for second opinions).

Finally, when doctors wished to re-use a photograph for purposes other than those initially discussed with the patients, the ability to anonymise the patient’s identity is an important factor for (not) asking for a new consent. When it is possible to anonymise the patient (Q 27), 26% of the doctors said they do not ask for a new consent (23% ask for written consent and 35% for oral consent, and 16% warn the patient about the situation). Conversely, when it is not possible to anonymise the patient (Q 28), only 5% of the participants said they do not ask for a new consent (51% ask for written consent and 31% for verbal consent, and 13% warn the patient).

When associating both questions, we conclude that, among those who stated they do not ask for a new consent when it is possible to anonymise the patient, only 17% said that they still did not ask for a consent when that anonymisation is not possible. The remaining 82.6% said they would alter their behaviour and obtain oral/written consent, or at least warn the patient when anonymisation proved to be unfeasible. This demonstrates that the ability to anonymise the identity is a significant factor (*p* < 0.001) regarding the renewal of informed consent when re-utilising photographs for other intentions.

## 4. Discussion

Our results showed that 93% of medical professionals in a tertiary referral and academic Portuguese hospital use photography in their workplace. This percentage is slightly lower compared to that of a previous study, which analysed US medical professionals in dermatology and reported usage of 99% [13]. This difference can be explained because dermatology heavily depends on visual diagnosis, and, in our questionnaire, doctors of numerous specialties were involved. Of our respondents, 85% use a personal device to capture medical photographs, which contrasts with patients’ 16–40% acceptance rate of the use of doctors’ personal devices [14,20,21], and thus showing that our current practices can negatively impact on doctor–patient relationships. We also found that 93% use a phone camera to take a medical photograph, which is consistent with a previous study [7]. About 2/3 of all doctors use medical photography monthly or weekly, with doctors in surgical or medical–surgical fields making more frequent usage than the remainder of their peers, often on a weekly or more frequent basis, thus demonstrating the high frequency of this practice amongst doctors. One study found that junior practitioners took more photographs [11], which is consistent with our results.

The most frequent reasons for usage that we found in our study were to document a patients’ clinical progress, to ask for a second opinion, for education, research, and publication, and for a presentation at a medical conference. Previous studies have shown the same most-frequent reasons in their results; some present percentages similar to ours [6,13,17], whereas others [10,11] present lower percentages but rank the categories similarly. The present study also found that younger doctors use photography more often to document a patient’s clinical progress and to ask for a second opinion than their senior colleagues.

The patient perception of medical photography was previously studied, and it was revealed that there is an acceptance rate of 85% for patient charting, 84% for second opinions with another professional, 82% to monitor disease progression with treatment, 70% for tele-dermatology, 63% for education, and 61% for research [14]. Thus, patients accept that their doctors use photography for the reasons mentioned above, which coincide with the use shown in our questionnaire.

The most frequently used software to send a medical photograph for a second opinion was the WhatsApp application (56%), followed by personal email (34%), which was congruent with previous studies [18,22]. In our study, 79% of respondents also answered that they had received a medical photograph, also mostly through WhatsApp (66%) and personal email (31%), which are similar percentages for the types of software used by the respondents to send the photographs in our study. Previous studies have shown that a doctor working in the public sector received significantly more photographs than those that solely work in the private sector [11], and that photographs were most commonly sent to request advice on diagnosis and treatment [10]. A previous report also found that 75% of their respondents had received a photograph from a patient [13], emphasising the importance of a safe transfer of files, not only between physicians, but also between a doctor and their patient.

Concerning patient consent, 71% of the physicians always asked for consent before taking a photograph, which is congruent with previous studies [10,13]. We also found that doctors favoured the usage of verbal consent instead of written consent when documenting a patients’ clinical progress, when asking for a second opinion, and when they wanted to use the photograph for educational purposes; these results were similar to those of a previous study [23]. Doctors indicated they used the combination of verbal and written consent more often when they wanted to use the photographs for research and publication, and to use the images in a medical conference presentation. It would also be important to further study the reasons that doctors use different types of consent for the different uses of photographs. A previous study found that 75% of the respondents believed acquiring verbal consent before taking a medical photograph was enough to ensure patients’ right to privacy [7]. However, another study noted that verbal consent frequently lacks information about the intended usage of the collected data [24]. A patient perception questionnaire showed that 91% of patients that have had a medical photograph taken recalled being asked permission to do so, with 75% providing verbal consent and 25% written consent. When written consent was obtained, 53% said they only read part of the consent form, 33% said they read the entire form, and 7% said they did not read the consent form at all [25]. Patients’ preferences for the form of providing consent were contradictory between studies, with one showing 40% of respondents indicated only verbal consent was needed and 60% indicating written consent was required [14]; another study demonstrated that 52% of respondents preferred verbal consent, 27% written consent, and 21% thought specific permission was unnecessary [25]. Further study into patient perceptions is important, to create guidelines for doctors in regard to medical photography that respect patients’ views on the subject.

When re-using a patient’s photograph for purposes other than those initially discussed with the patient, we demonstrated that the ability to anonymise or pseudo-anonymise the subject is a factor in the decision. According to another study, most patients are willing to consent to the re-usage of their medical photographs for education, with the study showing that comfort reached 90% when used in a one-on-one learner education, 80% in a large group for medical education, 73% for presentation at a national professional meeting, and 68% for publication in a medical journal or textbook [25]. However, it is important to note that doctors should still ask for consent to re-use the photographs in any context.

Our study also found that 58% of respondents stored medical photographs in the same device with which the photograph was taken, a value similar to that reported by others [6]. This was followed by storing in a personal device (38%), in a hospital device (11%), and in a personal cloud (11%). A previous study reported that 30% of their respondents had at least one medical photograph stored in their smartphones when the questionnaire was conducted [13]. It is important to note that patients may consider these practices a breach of their privacy, and various studies and guidelines have discussed the dangers of instant messaging applications such as WhatsApp [22] and cloud-based storage; many of these applications make it technically feasible for otherwise unauthorised individuals to access patient data [6,15,18,26,27]. It has also been shown that patients are more comfortable with medical photographs being stored directly inside their medical files [25]. It is important to note that physicians generally are not aware of or concerned about privacy and security regulations or guidelines because they believe the benefits to the patient and facilitation of medical treatment outweigh the obstacles related to compliance [22].

This study has some limitations. First, because it was conducted at a tertiary academic hospital and because of the response rate, its representativeness may be limited. Second, selection bias cannot be excluded, as this was a voluntary questionnaire and possible bias might have emerged related to the motivation to participate in the study. Third, a nonresponse bias may have also occurred because medical professionals may have chosen not to participate based on concerns relating to their use, or not, of digital photography and smartphones in the clinical setting. Moreover, women were only slightly under-represented (53.3% of the respondents) but older physicians were heavily under-represented in our study.

## 5. Conclusions

We found that the use of smartphones for medical photography is common among the hospital clinicians who responded to the questionnaire. These results also reveal that consent, documentation, and data storage security of clinical photographs are regularly handled sub-optimally. This study questioned numerous respondents of several specialties, showing “real world” data, and was performed in a teaching hospital following the implementation in the EU of GDPR. There is a need to reconcile smartphone technology advances and advantages with the current general and healthcare privacy laws [16], without disturbing the significant advance in collaboration, information flow, and, consequently, the improvement in patient care [28]. It is also important to note that security and safety of medical photographs can also be obtained when used with practice guidelines [29]. The authors feel, like others, that there is a need for secure applications (apps) [7,28,30] that can settle consent and privacy concerns, and be used efficiently in different contexts, such as at a patients’ bedside, during a medical consultation, or in the emergency room or operating theatre. It is also essential to create a consensus statement and implement practice guidelines for simple, safe, and secure capture of medical images, and their transfer, storage, and retrieval, involving the cooperative efforts of professional organisations and governance structures.

## Figures and Tables

**Table 1:** Sample characterisation.

	Total Sample (*n* = 257)
	*n* (%)
Female Gender	137 (53.3)
Age, years	
≤40	177 (70.2)
>40	75 (29.8)
Practitioner level	
Resident	116 (45.3)
Specialist	140 (54.7)
Type of medical specialty *	
Medical field	140 (55.8)
Medical-surgical	72 (28.7)
Surgical	39 (15.5)

* See Supplementary Material S2 for the specialties included in each group.

**Table 2:** Association of sociodemographic variables with questionnaire questions regarding the use of medical photography.

	Total Sample *n* (%)	Practitioner Level			Age		
		Resident	Specialist	*p*-Value	≤40 Years	>40 Years	*p*-Value
**Why do you take photographs of patients? (Q 4)**							
I do not use it on any occasion (Q 4.1)	18 (7.0)	5 (4.3)	13 (9.3)	0.121	7 (4.0)	11 (14.7)	**0.003**
To document the patient’s clinical progress (Q 4.2)	180 (70.0)	91 (78.4)	89 (63.6)	**0.01**	137 (77.4)	39 (52.0)	**<0.001**
To ask for a second opinion (Q 4.3)	181 (70.4)	95 (81.9)	85 (60.7)	**<0.001**	138 (78.0)	39 (52.0)	**<0.001**
For educational purposes (Q 4.4)	144 (56.0)	62 (53.4)	81 (57.9)	0.479	101 (57.1)	41 (54.7)	0.726
For research and publication (Q 4.5)	166 (64.6)	81 (69.8)	85 (60.7)	0.128	122 (68.9)	39 (52.0)	0.011
To present at a medical conference (Q 4.6)	175 (68.1)	87 (75.0)	87 (62.1)	0.028	130 (73.4)	40 (53.3)	**0.002**
**How often do you take photographs of patients? (Q 5)**							
Weekly or more frequently	105 (45.3)	42 (38.2)	63 (52.1)	0.034	74 (44.0)	29 (49.2)	0.498
Monthly of less frequently	127 (54.7)	68 (61.8)	58 (47.9)		94 (56.0)	30 (50.8)	
**What kind of device do you use to take photographs? (Q 8)**							
Personal device	191 (84.5)	100 (91.7)	90 (77.6)	**0.003**	148 (89.2)	40 (71.4)	**0.001**
Other (personal only for professional use/institutional)	35 (15.5)	9 (8.3)	26 (22.4)		18 (10.8)	16 (28.6)	
**Where do you document oral/written consent? (Q 13)**							
I don’t document anywhere	48 (20.2)	21 (19.4)	26 (20.2)	0.902	36 (21.8)	11 (15.9)	0.466
In personal documents	24 (10.1)	10 (9.3)	14 (10.9)		18 (10.9)	6 (8.7)	
Other (patient’s clinical record/with the photograph)	166 (69.7)	77 (71.3)	89 (69.0)		111 (67.3)	52 (75.4)	
**I have received photographs for a second opinion (Q 23)**	198 (78.6)	90 (78.3)	107 (78.7)	0.936	143 (82.2)	53 (71.6)	0.062
	**Total Sample *n* (%)**	**Type of Specialty**					
		**Medical**	**Medical-Surgical**	**Surgical**	***p*** **-Value**		
**Why do you take photographs of patients? (Q 4)**							
I do not use it on any occasion (Q 4.1)	18 (7.0)	14 (10.0)	2 (2.8)	1 (2.6)	0.103		
To document the patient’s clinical progress (Q 4.2)	180 (70.0)	87 (62.1)	58 (80.6)	31 (79.5)	**0.008**		
To ask for a second opinion (Q 4.3)	181 (70.4)	94 (67.1)	52 (72.2)	32 (82.1)	0.185		
For educational purposes (Q 4.4)	144 (56.0)	77 (55.0)	46 (63.9)	18 (46.2)	0.182		
For research and publication (Q 4.5)	166 (64.6)	90 (64.3)	48 (66.7)	25 (64.1)	0.936		
To present at a medical conference (Q 4.6)	175 (68.1)	87 (62.1)	54 (75.0)	29 (74.4)	0.104		
**How often do you take photographs of patients? (Q 5)**							
Weekly or more frequently	105 (45.3)	41 (32.8)	41 (62.1)	21 (56.8)	**<0.001**		
Monthly of less frequently	127 (54.7)	84 (67.2)	25 (37.9)	16 (43.2)			
**What kind of device do you use to take photographs? (Q 8)**							
Personal device	191 (84.5)	102 (82.9)	53 (84.1)	32 (91.4)	0.466		
Other (personal only for professional use/institutional)	35 (15.5)	21 (17.1)	10 (15.9)	3 (8.6)			
**Where do you document oral/written consent? (Q 13)**							
I don´t document anywhere	48 (20.2)	15 (11.5)	20 (29.0)	13 (38.2)	**0.002**		
In personal documents	24 (10.1)	17 (13.0)	4 (5.8)	3 (8.8)			
Other (patient’s clinical record/with the photograph)	166 (69.7)	99 (75.6)	45 (65.2)	18 (52.9)			
**I have received photographs for a second opinion (Q 23)**	198 (78.6)	100 (73.0)	61 (84.7)	35 (92.1)	0.015		

Values in bold are ones with a *p* ≤ 0.01.

**Table 3:** Types of consent.

	None	Only Verbal Consent	Only Written Consent	Both Verbal and Written Consent
**Why do you take photographs of patients? (Q 4)**				
To document the patient’s clinical progress (Q 4.2)	37 (20.6)	120 (66.7)	8 (4.4)	15 (8.3)
To ask for a second opinion (Q 4.3)	43 (23.8)	126 (69.6)	3 (1.7)	9 (5.0)
For educational purposes (Q 4.4)	39 (27.1)	72 (50.0)	9 (6.3)	24 (16.7)
For research and publication (Q 4.5)	20 (12.0)	31 (18.7)	21 (12.7)	94 (56.6)
To present at a medical conference (Q 4.6)	33 (18.9)	49 (28.0)	21 (12.0)	72 (41.1)

Types of consent (Q 11/12) for the five most answered reasons for the use of medical photography (Q 4).

## Data Availability

The data that support the findings of this study are not openly available due to privacy and are available from the corresponding author upon reasonable request.

## Supplementary Materials

The following supporting information can be downloaded at: https://www.mdpi.com/article/10.3390/ijerph19127304/s1, Supplementary Material S1: Medical Photography Questionnaire: A translated copy of the distributed questionnaire.; Supplementary Material S2: Medical field groups: A table showing discrimination of categories of medical specialties for the study.

## Abbreviations

Q: question; SMS: Short Message Service; EU: European Union; GDPR: General Data Protection Regulation.
