# Supplementary material for: Medical Photography Usage Amongst Doctors at a Portuguese Hospital
Source: Int J Environ Res Public Health. 2022 Jun 14;19(12):7304. doi: 10.3390/ijerph19127304 (PMC9224246; doi:10.3390/ijerph19127304)
Supplement: Supplementary file 1 [file ijerph-19-07304-s001.zip › ijerph-1681043-SM.pdf]

Supplementary Material S1
Medical Photography Questionnaire

1. Medical Specialty: ______________________
    - ☐ Specialist
    - ☐ Resident

2. Gender
    - ☐ Female
    - ☐ Male

3. Age:
    - ☐ 20-40 years old
    - ☐ 41-60 years old
    - ☐ 61 years old or older

4. When you take a photograph/record a video of a patient, why do you do it? (fill all that apply)
    - ☐ I do not use it on any occasion
    - ☐ To document the patients' clinical progress
    - ☐ Ask a colleague for a second opinion
    - ☐ For educational purposes
    - ☐ Research and publication
    - ☐ To present at a medical conference
    - ☐ Register a biopsy site
    - ☐ Document wound appearance after surgery
    - ☐ Document a surgical procedure in the operating room
    - ☐ For legal purposes
    - ☐ For pathology reasons
    - ☐ For patient motivation, instruction and advisement of the patient
    - ☐ Other: ____

If you selected "I do not use it on any occasion", only answer the questions with an *

5. How often do you take photographs of patients?
    - ☐ Several times a day
    - ☐ Daily
    - ☐ Weekly
    - ☐ Monthly
    - ☐ Annually
    - ☐ Never

6. How often do you record videos of patients?
    - ☐ Several times a day
    - ☐ Daily
    - ☐ Weekly
    - ☐ Monthly
    - ☐ Annually
    - ☐ Never

7. With which device do you take photographs/record videos of patients'?
    - ☐ DSLR camera
    - ☐ Compact camera
    - ☐ Phone camera
    - ☐ Clinical photography application (If so, which: ____________________________)
    - ☐ Other: __________

8. The device is:
    - ☐ Personal
    - ☐ Personal only for professional use
    - ☐ Institutional

9. If you answered on question 7 to phone camera, which one do you use and what model (if you remember)
    - ☐ iPhone
    - ☐ Samsung

- ☐ Huawei
- ☐ Xiaomi
- ☐ OnePlus
- ☐ Blackberry
- ☐ LG
- ☐ Other: ________________

*10. How frequently do/*would you ask for a patient consent for taking a photograph/recording a video?

- ☐ Always before taking it
- ☐ Always after taking it
- ☐ Often before taking it
- ☐ Often after taking it
- ☐ When I remember
- ☐ Rarely
- ☐ Never

*11. In which situations do/*would you ask for a verbal consent? (fill all that apply)

- ☐ To document the patients' clinical progress
- ☐ Ask for a second opinion
- ☐ For educational purposes
- ☐ Research and publication
- ☐ To present at a medical conference
- ☐ Register a biopsy site
- ☐ Document wound appearance after surgery
- ☐ Document a surgical procedure
- ☐ For legal purposes
- ☐ For pathology reasons
- ☐ For patient motivation, instructive and advisement of the patient
- ☐ Other: ___________

*12. In which situations do/*would you ask for a written consent? (fill all that apply)

- ☐ To document the patients' clinical progress
- ☐ Ask for a second opinion
- ☐ For educational purposes
- ☐ Research and publication
- ☐ To present at a medical conference
- ☐ Register a biopsy site
- ☐ Document wound appearance after surgery
- ☐ Document a surgical procedure
- ☐ For legal purposes
- ☐ For pathology reasons
- ☐ For patient motivation, instructive and advisement of the patient
- ☐ Other: ________

*13. Where do/*would you document the consent?

- ☐ Patients clinical record (digital or physical)
- ☐ With the photograph
- ☐ In personal documents
- ☐ I don't (*would) document anywhere

*14. What do/*would you say to the patient before obtaining his/her consent? (fill all that apply)

- ☐ Reason for medical photography
- ☐ Ask in what contexts it can be used
- ☐ Where they are stored
- ☐ Who has access
- ☐ Consent can be withdrawn at any time without compromise in the patient-doctor relationship, and that photographs will be deleted if the patient wishes
- ☐ How long the consent lasts

15. What measures do (*would) you take when taking photographs of a patient? (fill all that apply)

- ☐ Light
- ☐ Background
- ☐ Center subject
- ☐ Show anatomical landmarks or scales for posterior reference
- ☐ Focus
- ☐ Image Sharpness
- ☐ Realistic colour
- ☐ White balance
- ☐ Not include unnecessary elements

☐ Does not apply

16. What measures do (*would) you take when recording a video of a patient? (fill all that apply)
- ☐ Light
- ☐ Background
- ☐ Center subject
- ☐ Show anatomical landmarks or scales for posterior reference
- ☐ Focus
- ☐ Image Sharpness
- ☐ Realistic colour
- ☐ White balance
- ☐ Not include unnecessary elements
- ☐ Does not apply

17. Do you use a program to enhance the photograph quality or to edit videos of patients? (If yes, which program and what do you do?)
- ☐ For photographs: ____
- ☐ For videos: ____
- ☐ I do not use

18. Where do you store patients photographs/videos? (fill all that apply)
- ☐ On the device where the recording was obtained
- ☐ Personal device
- ☐ Personal device only for professional use
- ☐ Institutional device
- ☐ Patients' digital record
- ☐ Specific institutional server
- ☐ Personal Cloud (Google Drive, iCloud, Dropbox…)
- ☐ Personal Cloud only for professional use (Google Drive, iCloud, Dropbox…)
- ☐ Personal hard-drive or equivalent
- ☐ Personal hard-drive or equivalent only for professional use
- ☐ Institutional hard-drive or equivalent
- ☐ Does not apply

19. How do you transfer the recordings'? (fill all that apply)
- ☐ Personal email
- ☐ Institutional email
- ☐ Cable that links to a computer
- ☐ Cable that uses the computer as a bridge to a central safe server
- ☐ Download from an unknown Wi-Fi
- ☐ Download from a secure Wi-Fi
- ☐ Secure transfer application
- ☐ Bluetooth
- ☐ Does not apply

20. When do you delete the recordings from the device?
- ☐ Immediately after storing them in another place that is not the device
- ☐ When I remember after storing them in another place that is not the device
- ☐ It always stays in the device
- ☐ Does not apply

21. What measures do you take in order to ensure the devices security? (fill all that apply)

- ☐ Password (6 or more digits)
- ☐ Encryption
- ☐ 2-factor authentication
- ☐ Stored in a safe place with restricted access
- ☐ Cloud backup (Google Drive, iCloud, Dropbox…)
- ☐ Keep software updated (for security bugs)
- ☐ Remote deletion of the device content
- ☐ Connection to safe Wi-Fi
- ☐ GPS, date, and hour disabled for photographs
- ☐ Unknown, device security handled by the institution
- ☐ None of the above/unknown

22. If the photographs/videos are used to ask for a second opinion, how are they sent? (fill all that apply)
- ☐ Personal email
- ☐ Institutional email
- ☐ SMS
- ☐ WhatsApp
- ☐ Viber
- ☐ Messenger
- ☐ Secure medical photography app
- ☐ Does not apply
- ☐ Other: ________________________

*23. Have you ever received a photograph/video of a patient for your second opinion?
- ☐ Yes
- ☐ No

*24. If you answered yes on question 23, how frequently?
- ☐ Several times a day
- ☐ Daily
- ☐ Weekly
- ☐ Monthly
- ☐ Annually
- ☐ Never

*25. If you answered yes on 23, how are they sent? (fill all that apply)
- ☐ Personal email
- ☐ Institutional email
- ☐ SMS
- ☐ WhatsApp
- ☐ Viber
- ☐ Messenger
- ☐ Secure medical photography app
- ☐ Does not apply
- ☐ Other: ______________________

*26. Have you ever used photographs/videos of patients in any of the following occasions? (fill all that apply)
- ☐ Digital or physical publication
- ☐ Medical congress presentation
- ☐ Education
- ☐ Publicity

☐ Never used

*27. If you wanted to use the photograph/video for means that were not initially discussed with the patient and it is possible to make the recording anonymous, what do/*would you do?

☐ Warn the patient that the recording will be used
☐ Ask for verbal consent
☐ Ask for written consent
☐ I do/*would not ask for consent

*28. If you wanted to use the photograph/video for means that were not initially discussed with the patient and it is not possible to make the recording anonymous, what do/*would you do?

☐ Warn the patient that the recording will be used
☐ Ask for verbal consent
☐ Ask for written consent
☐ I do/*would not ask for consent

*29. Have you ever taken a photograph of a patients' clinical record or of medical exams with a personal device?

☐ Yes
☐ No

*30. Have you ever shared the photograph mentioned in question 29?

☐ Yes
☐ No

Comments:

Supplementary Material S2

Discrimination of categories: 35 specialties included

Medical specialties	Medico-surgical specialties	Surgical specialties
Anaesthesiology	Dermatology	Angiology and Vascular Surgery
Anatomical Pathology	Obstetrics and Gynaecology	Cardiothoracic Surgery
Cardiology	Ophthalmology	General Surgery
Clinical Pathology	Otorhinolaryngology	Maxillofacial Surgery
Clinical Pharmacology	Stomatology	Neurosurgery
Endocrinology	Urology	Orthopaedic surgery
Family Medicine		Paediatric Surgery
Gastroenterology		Plastic Surgery
Haematology		
Infectious Diseases		
Intensive Care Medicine		
Internal Medicine		
Medical Genetics		
Nephrology		
Neurology		
Oncology		
Paediatrics		
Physical Medicine and Rehabilitation		
Pneumology		
Psychiatry		
Rheumatology		
